# Supplementary material for: Successful Clearance of 300 Day SARS-CoV-2 Infection in a Subject with B-Cell Depletion Associated Prolonged (B-DEAP) COVID by REGEN-COV Anti-Spike Monoclonal Antibody Cocktail
Source: Viruses. 2021 Jun 23;13(7):1202. doi: 10.3390/v13071202 (PMC8310246; doi:10.3390/v13071202)
Supplement: Supplementary file 1 [file viruses-13-01202-s001.zip › B-DEAP COVID Suppl Tables.pptx]

## Slide 1
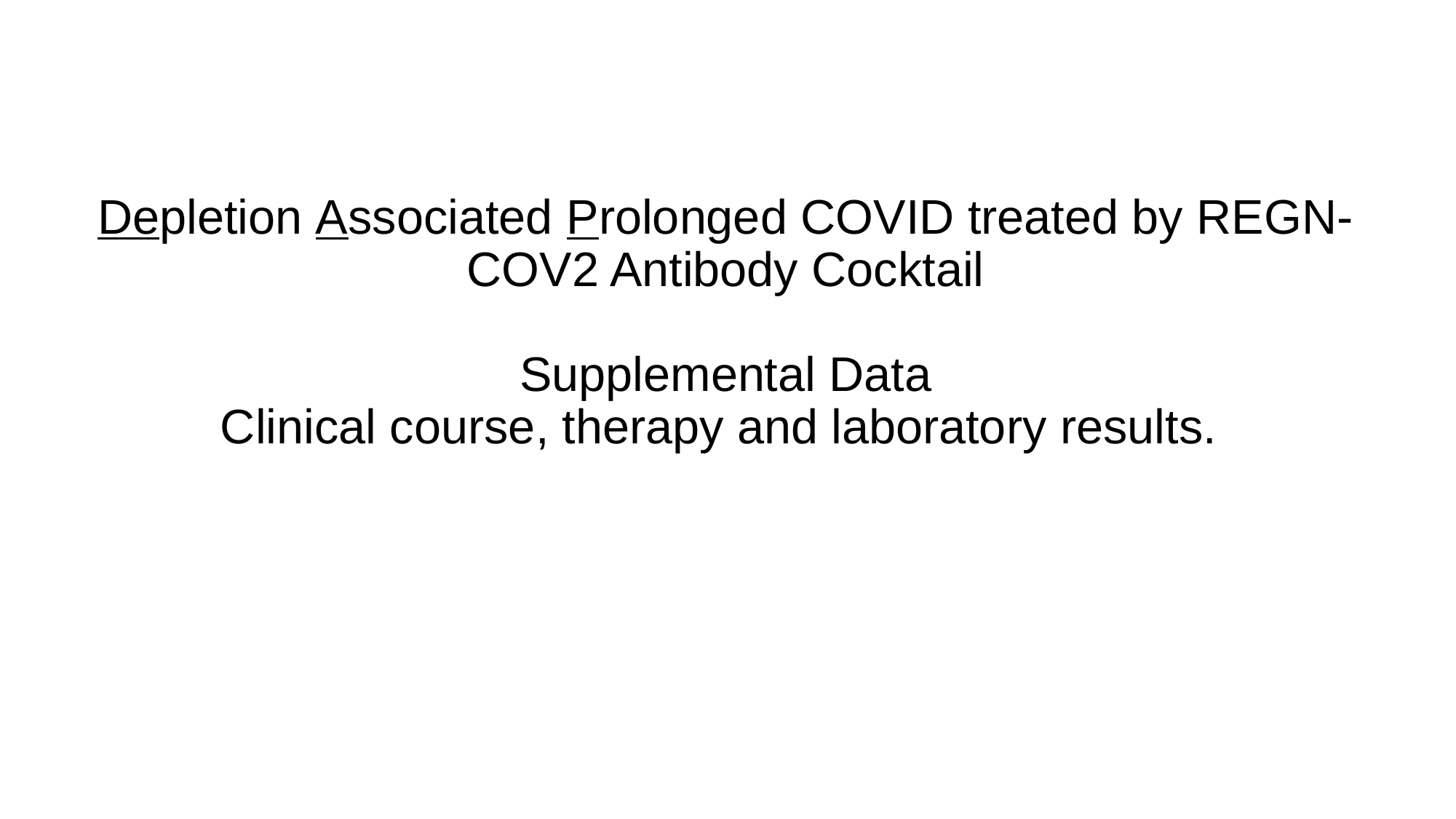

# Depletion Associated Prolonged COVID treated by REGN-COV2 Antibody Cocktail Supplemental Data Clinical course, therapy and laboratory results.

## Slide 2
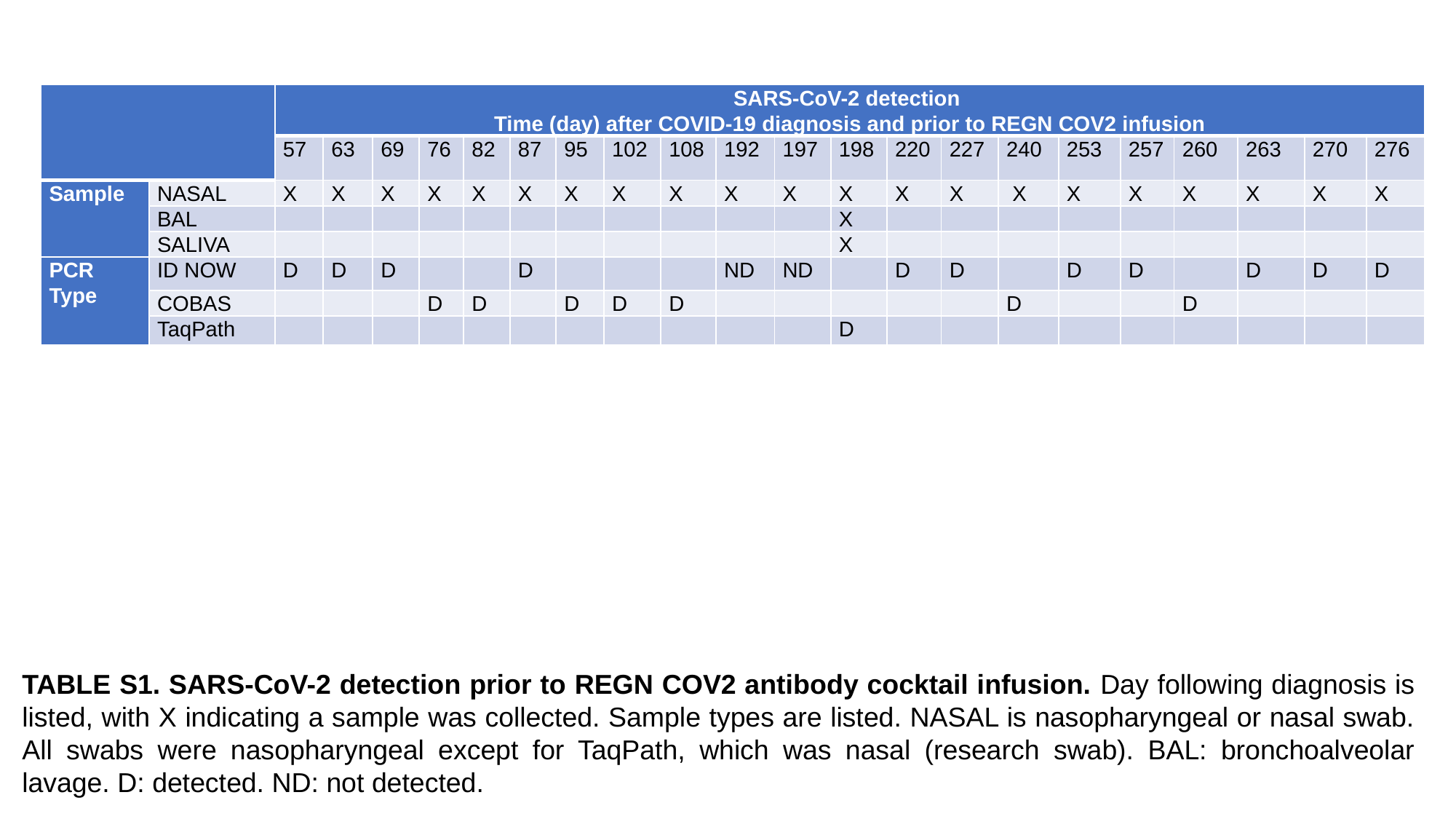

| | | SARS-CoV-2 detection Time (day) after COVID-19 diagnosis and prior to REGN COV2 infusion | | | | | | | | | | | | | | | | | | | | |
| --- | --- | --- | --- | --- | --- | --- | --- | --- | --- | --- | --- | --- | --- | --- | --- | --- | --- | --- | --- | --- | --- | --- |
| | | 57 | 63 | 69 | 76 | 82 | 87 | 95 | 102 | 108 | 192 | 197 | 198 | 220 | 227 | 240 | 253 | 257 | 260 | 263 | 270 | 276 |
| Sample | NASAL | X | X | X | X | X | X | X | X | X | X | X | X | X | X | X | X | X | X | X | X | X |
| | BAL | | | | | | | | | | | | X | | | | | | | | | |
| | SALIVA | | | | | | | | | | | | X | | | | | | | | | |
| PCR Type | ID NOW | D | D | D | | | D | | | | ND | ND | | D | D | | D | D | | D | D | D |
| | COBAS | | | | D | D | | D | D | D | | | | | | D | | | D | | | |
| | TaqPath | | | | | | | | | | | | D | | | | | | | | | |
TABLE S1. SARS-CoV-2 detection prior to REGN COV2 antibody cocktail infusion. Day following diagnosis is listed, with X indicating a sample was collected. Sample types are listed. NASAL is nasopharyngeal or nasal swab. All swabs were nasopharyngeal except for TaqPath, which was nasal (research swab). BAL: bronchoalveolar lavage. D: detected. ND: not detected.

## Slide 3
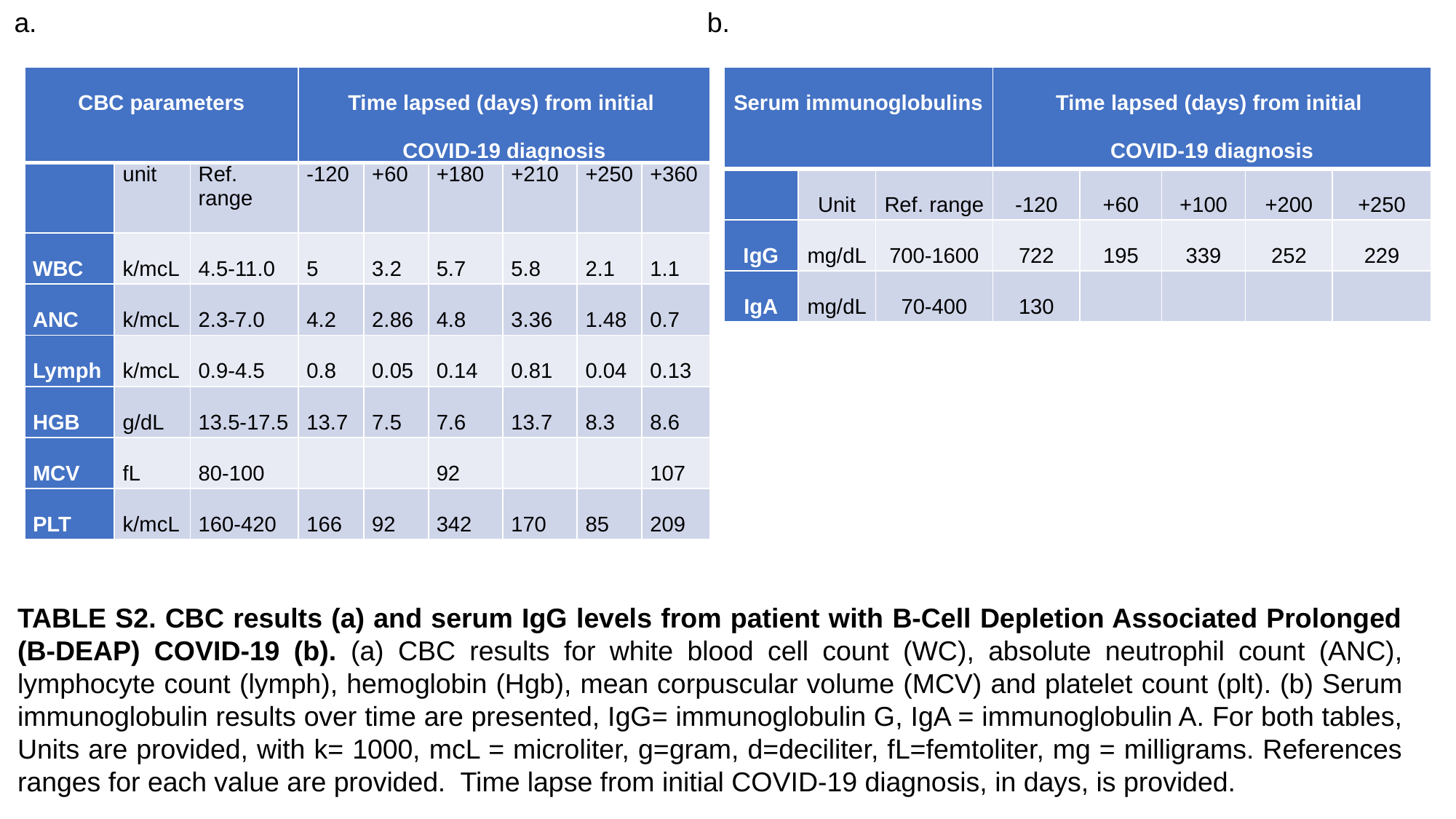

a.						 b.
| CBC parameters | | | Time lapsed (days) from initial COVID-19 diagnosis | | | | | |
| --- | --- | --- | --- | --- | --- | --- | --- | --- |
| | unit | Ref. range | -120 | +60 | +180 | +210 | +250 | +360 |
| WBC | k/mcL | 4.5-11.0 | 5 | 3.2 | 5.7 | 5.8 | 2.1 | 1.1 |
| ANC | k/mcL | 2.3-7.0 | 4.2 | 2.86 | 4.8 | 3.36 | 1.48 | 0.7 |
| Lymph | k/mcL | 0.9-4.5 | 0.8 | 0.05 | 0.14 | 0.81 | 0.04 | 0.13 |
| HGB | g/dL | 13.5-17.5 | 13.7 | 7.5 | 7.6 | 13.7 | 8.3 | 8.6 |
| MCV | fL | 80-100 | | | 92 | | | 107 |
| PLT | k/mcL | 160-420 | 166 | 92 | 342 | 170 | 85 | 209 |
| Serum immunoglobulins | | | Time lapsed (days) from initial COVID-19 diagnosis | | | | |
| --- | --- | --- | --- | --- | --- | --- | --- |
| | Unit | Ref. range | -120 | +60 | +100 | +200 | +250 |
| IgG | mg/dL | 700-1600 | 722 | 195 | 339 | 252 | 229 |
| IgA | mg/dL | 70-400 | 130 | | | | |
TABLE S2. CBC results (a) and serum IgG levels from patient with B-Cell Depletion Associated Prolonged (B-DEAP) COVID-19 (b). (a) CBC results for white blood cell count (WC), absolute neutrophil count (ANC), lymphocyte count (lymph), hemoglobin (Hgb), mean corpuscular volume (MCV) and platelet count (plt). (b) Serum immunoglobulin results over time are presented, IgG= immunoglobulin G, IgA = immunoglobulin A. For both tables, Units are provided, with k= 1000, mcL = microliter, g=gram, d=deciliter, fL=femtoliter, mg = milligrams. References ranges for each value are provided. Time lapse from initial COVID-19 diagnosis, in days, is provided.

## Slide 4
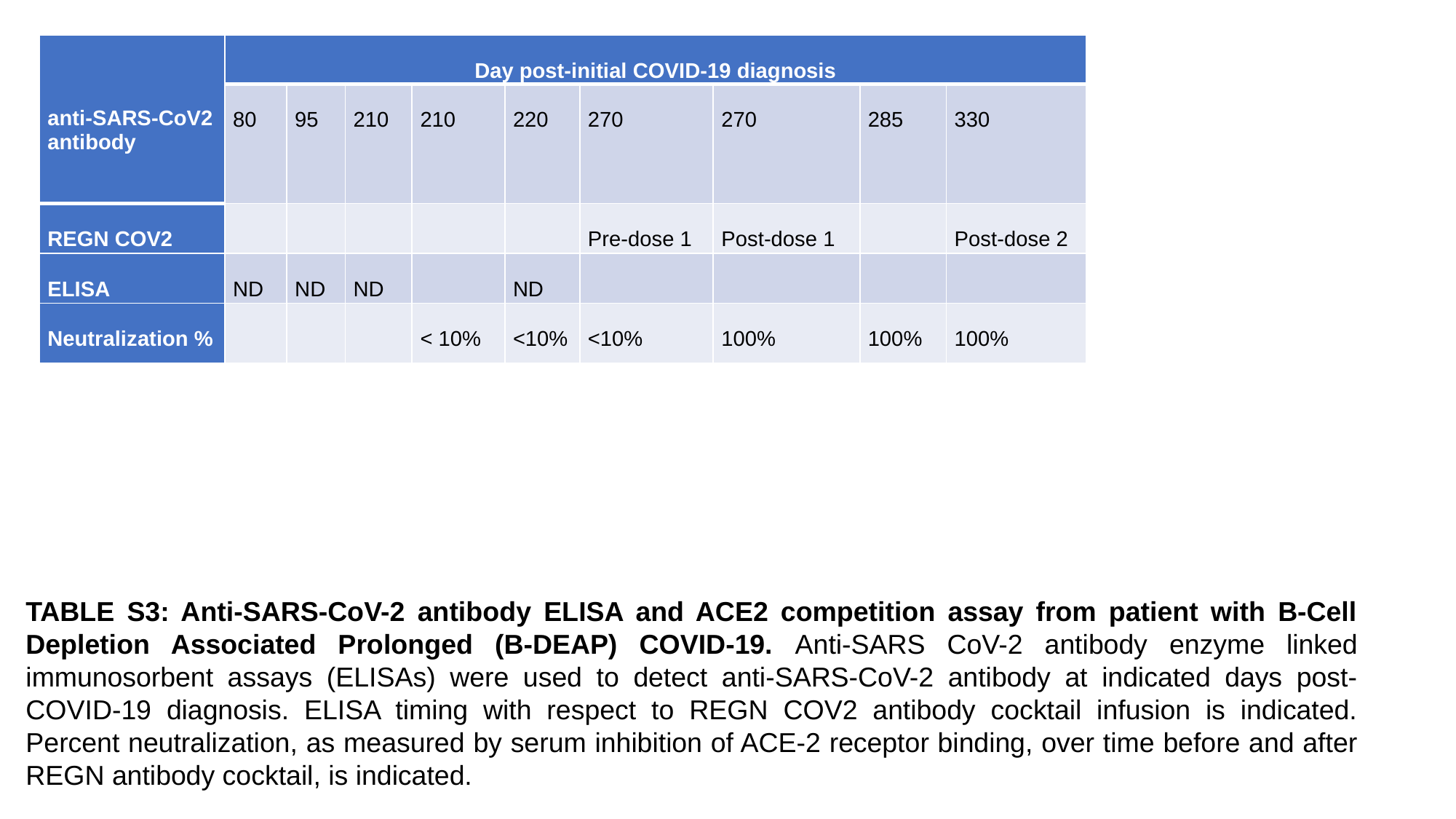

| anti-SARS-CoV2 antibody | Day post-initial COVID-19 diagnosis | | | | | | | | |
| --- | --- | --- | --- | --- | --- | --- | --- | --- | --- |
| | 80 | 95 | 210 | 210 | 220 | 270 | 270 | 285 | 330 |
| REGN COV2 | | | | | | Pre-dose 1 | Post-dose 1 | | Post-dose 2 |
| ELISA | ND | ND | ND | | ND | | | | |
| Neutralization % | | | | < 10% | <10% | <10% | 100% | 100% | 100% |
TABLE S3: Anti-SARS-CoV-2 antibody ELISA and ACE2 competition assay from patient with B-Cell Depletion Associated Prolonged (B-DEAP) COVID-19. Anti-SARS CoV-2 antibody enzyme linked immunosorbent assays (ELISAs) were used to detect anti-SARS-CoV-2 antibody at indicated days post-COVID-19 diagnosis. ELISA timing with respect to REGN COV2 antibody cocktail infusion is indicated. Percent neutralization, as measured by serum inhibition of ACE-2 receptor binding, over time before and after REGN antibody cocktail, is indicated.

## Slide 5
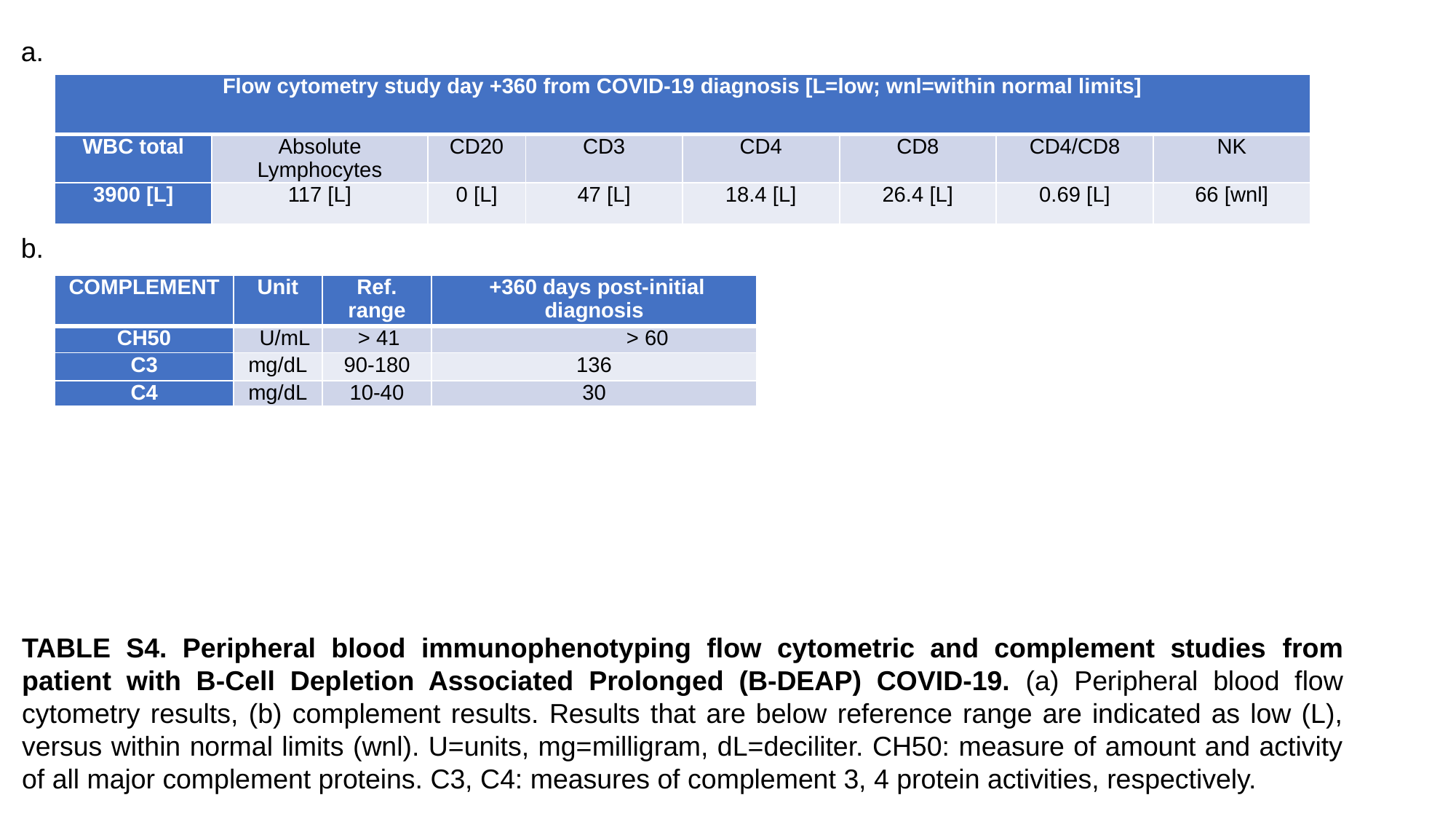

a.
b.
| Flow cytometry study day +360 from COVID-19 diagnosis [L=low; wnl=within normal limits] | | | | | | | |
| --- | --- | --- | --- | --- | --- | --- | --- |
| WBC total | Absolute Lymphocytes | CD20 | CD3 | CD4 | CD8 | CD4/CD8 | NK |
| 3900 [L] | 117 [L] | 0 [L] | 47 [L] | 18.4 [L] | 26.4 [L] | 0.69 [L] | 66 [wnl] |
| COMPLEMENT | Unit | Ref. range | +360 days post-initial diagnosis |
| --- | --- | --- | --- |
| CH50 | U/mL | > 41 | > 60 |
| C3 | mg/dL | 90-180 | 136 |
| C4 | mg/dL | 10-40 | 30 |
TABLE S4. Peripheral blood immunophenotyping flow cytometric and complement studies from patient with B-Cell Depletion Associated Prolonged (B-DEAP) COVID-19. (a) Peripheral blood flow cytometry results, (b) complement results. Results that are below reference range are indicated as low (L), versus within normal limits (wnl). U=units, mg=milligram, dL=deciliter. CH50: measure of amount and activity of all major complement proteins. C3, C4: measures of complement 3, 4 protein activities, respectively.
