# Supplementary material for: Successful Clearance of 300 Day SARS-CoV-2 Infection in a Subject with B-Cell Depletion Associated Prolonged (B-DEAP) COVID by REGEN-COV Anti-Spike Monoclonal Antibody Cocktail
Source: Viruses. 2021 Jun 23;13(7):1202. doi: 10.3390/v13071202 (PMC8310246; doi:10.3390/v13071202)
Supplement: Supplementary file 1 [file viruses-13-01202-s001.zip › Brief report CSS 94 Materials Methods AD edits 4-21-2021 1120AM (1).pdf]

CSS #94 Brief report Materials and Methods

Sample processing protocols:

Plasma prepared from anticoagulated fresh blood draw and residual samples were frozen and stored at -80C in aliquots of 200mcL.

SEROLOGY TESTS

1- ReSARS® CoV-2 N-protein and S-RBD IgG ELISA kits for RUO, Zalgen Labs, LLC: are formatted as direct ELISAs with recombinant antigens coated directly on to 96-well microtiter plates (Nunc) and stabilized with proprietary block solution. The assays are semi-quantitative with convalescent COVID-19 donor plasma (lyophilized) provided to prepare a Reference Curve. An additional COVID-19 donor plasma (lyophilized) is provided as a Positive Control and bulk normal human plasma (lyophilized) is provide as a Negative Control. After reconstituting the Reference and Controls with purified water, the Reference, Controls and Human plasma samples (K3EDTA) are diluted 1:101 in Sample Diluent (10µL:1000µL). The Reference is further diluted 4-fold to prepare five-point Reference Curve. The Reference Curve, Controls, and Samples are added to duplicate, designated microwells at 100µL/well and incubated at ambient temperature (18 - 30°C) for 30 minutes. Two wells of just Sample Diluent are included as a Reagent Blank. Following incubation, the plates are washed 4 x 300µL/well with PBS-Tween wash buffer. For detection of N-protein or S-RBD specific IgG, affinity purified Goat anti-Human IgG HRP conjugate reagent is added to the microwells at 100µL/well and incubated at ambient temperature for 30 minutes. PBS-Tween wash is repeated to remove conjugate reagent. TMB Substrate (Moss) is added to microwells (100µL/well) for 10 minutes at ambient temperature. Substrate color development is stopped with the addition of 2% methane sulfonic acid Stopping Solution. Microwell plates are read at 450nm with 650nm reference. Gen5 program is used to subtract mean 450nm-650nm followed by Reagent Blank subtraction. Specific IgG (units/mL) are calculated from a 4- parameter logistic fit of the Reference Curve. The valuation of the Reference plasma is based on a 0-100 scale with 100 units/mL equivalent to maximum clinical titer. Negative cut-off is equivalent to 2 units/mL based on Mean + 2.5X Standard Deviation of +100 Pre-COVID US normal plasmas.

2- SARS-CoV-2 anti-S (HEXAPRO) ELISA for RUO (Dr Robinson's Lab):

A plasmid for expression of stable pre-fusion trimeric SARS CoV-2 spike protein, similar to that described by Wrapp et al (PMID: 32511295) was kindly provided by Kate Hastie (La Jolla Institute for Immunology). Recombinant stabilized spike protein was produced and purified as described by Dan et al. (PMID: 33408181). Wells of 96-well ELISA plates (Costar, Easy Wash) were coated for 1 hour at room temperature with this SARS CoV-2 spike protein (500 ng/well in 100 mM sodium bicarbonate buffer). Wells were washed X 5 and blocked for 1 hour with 0.5%Tween, 5% dry milk, 4% whey, 10%FBS in 1x PBS at +37°C. Sera or plasma samples diluted to 1:100 in the same buffer were incubated in antigen coated and uncoated wells for 1 h at room temperature. Bound IgG was detected with peroxidase-conjugated goat anti-IgG (Jackson Immunoresearch) and color was developed with TMB-peroxidase as described (PMID: 27161536). Net OD values at 450 nm were calculated by subtracting background OD readings from OD readings with spike protein. A cut off net OD value of 0.451 was calculated based on testing of >100 pre-covid serum samples.

3- COVID-19 ACE2 Competition Assay® (V-PLEX SARS-CoV-2 Panel 2), Meso Scale Discovery, for RUO:

This multiplexed solid-phase chemi-luminescence assay allows the simultaneous detection of IgG binding to four SARS-CoV-2 antigens and the quantification of antibody-induced ACE-2 binding inhibition. The latter is an ELISA based pseudo-neutralization assay, detecting antibodies able to block the binding of angiotensin-converting enzyme 2 (ACE2) to the SARS-CoV-2 Spike and S1 RBD antigens. Plates were blocked and washed, serial dilution of assay calibrator (COVID-19 neutralizing antibody; monoclonal antibody against S protein; 200 unit/mL), control and plasma samples (10 µL) diluted 1 in 100 in assay diluent were added to the plates. Following sample incubation an 0.25 µg/mL solution of MSD SULFO-TAG™ conjugated ACE-2 was added after which plates were read. The assay was performed with the Meso QuickPlex SQ120 on a 10-spot 96 well microplate. Data was extrapolated from a calibration curve and calculated as percent inhibition normalized by a blank sample or as concentration of neutralizing SARS-CoV/SARS-CoV-2 Spike monoclonal antibody. One unit/mL of concentration of calibrator corresponds to neutralizing activity of 1 µg/mL monoclonal antibody to SARS-CoV/SARS-Cov-2 Spike protein.

$\%\ inhbition = [(1 - average\ sample\ signal)/average\ signal\ of\ Calibrator\ 8] * 100.$

MOLECULAR TESTS:

1- CDC Emergency Use Authorization (EUA) 2019-nCoV Real Time qRT-PCR Panel protocol, A. Smither and Dr. Garry's Lab. for RUO:

Samples: Research swabs, saliva samples and BAL collected prior to REGN infusion were processed with this qRT-PCR protocol.

Sample RNA extraction was performed with QIAmp Viral RNA Mini Kit (Qiagen) according to the manufacturer's instructions.

Primers: The presence of SARS-CoV-2 Nucleocapsid (NP) was assessed using 2 sets of primers designed to target two portions of the NP gene, and a human gene RNAseP (RP).

**Primer/probe mix**	**Sequence**	
**2019-nCoV_N1**	Forward	5'-GACCCCAAAATCAGCGAAAT-3'
	Reverse	5'-TCTGGTTACTGCCAGTTGAATCTG-3'
	Probe	5'- FAM-ACCCCGCAT/ZEN/TACGTTTGGTGGACC-3IABkFQ-3'
**2019-nCoV_N2**	Forward	5'-TTACAAACATTGGCCGCAAA -3'
	Reverse	5'-GCGCGACATTCCGAAGAA -3'
	Probe	5'-FAM-ACAATTTGC/ZEN/CCCCAGCGCTTCAG-3IABkFQ-3'
**Hu RNAseP (RP)**	Forward	5'-AGATTTGGACCTGCGAGCG -3'
	Reverse	5'-GAGCGGCTGTCTCCACAAGT -3'
	Probe	5'- FAM-TTC TGA CCT /ZEN/ GAA GGC TCT GCG CG-3IABkFQ-3'

Reaction mix: 8.5µL nuclease-free water, 1.5µL combined primer/probe mix (Integrated DNA Technologies), 5µL 4x TaqPath 1-Step RT-qPCR Master Mix, CG (Applied Biosystems), and 5µL of RNA (extract or standard) in a final volume of 20mcL.

Each primer/probe set was run as a singlet for a total of three reactions/specimen.

Amplification: cycling parameters programed as 25°C for 2 minutes, 50°C for 15 minutes, 95°C for 2 minutes, and 40 cycles of 95°C for 3 seconds and 55°C for 30 seconds, in a QuantStudio 3 Real-Time PCR System (Applied Biosystems). **Calibrator:** Viral load was assessed using an N1-specific ssRNA standard dilution series ranging from 10^8-10^1 copies (kind gift of Chris. Monjure). **Analysis:** A specimen was considered positive if all targets, viral (N1; N2), and human (RP) had a Ct <35.

2- Applied Biosystems TaqPath COVID-19 Combo Kit, Dr. Tian's Lab., for IVD use:

Research swabs collected immediately prior to and after REGN MoAb infusions were processed in the Molecular Laboratory of the Tulane Department of Pathology (Dr Tian, MD., PhD. Director). The laboratory is certified under CLIA and accredited by CAP to perform high-complexity testing. The test is approved under FDA Emergency Use Authorization (EUA). RNA extraction was performed using the KingFisher Flex automated extractor. Extraction required a starting sample volume of 200ul of the swab initial material and 10mcl of the final 50mcl elution volume was used per RT-PCR reaction. The SARS-CoV-2 RT-PCR was performed with the Thermo-Fisher TaqPath COVID-19 Combo Kit after manufacturer's recommendations. For amplification, we used the QS5 QuantStudio Real-time PCR system, with a total of 40 cycles. A cut-off Ct value set at 37 cycles was used to set the detection of the viral genes N, ORF1ab and S.

3- SARS-CoV-2 genome sequencing (Andersen Lab. Scripps Research Institute), for RUO:

Samples with an N1 Ct<30 (correlating to ~500 copies of virus/µL) were selected for amplicon sequencing. Samples' viral RNA was shipped to Scripps Research Institute. All samples passed the **required QC**, consisting of RNA concentration measurement (Qubit) and fragment size evaluation (TapeStation) before sequencing. **Virus sequencing** was performed using NextSeq. **For analysis**, sequence reads were aligned to a hCoV-19 reference sequence and consensus sequences generated using iVar.
